# Use of bioluminescence measurements for detection of artificial demineralization adjacent to orthodontic brackets

**DOI:** 10.1007/s00056-021-00341-y

**Published:** 2021-07-30

**Authors:** Anahita Jablonski-Momeni, Janine Sambale, Laura Gaerttner, Romy Nothelfer, Heike Korbmacher-Steiner

**Affiliations:** Dental School, Department of Orthodontics, Philipps University of Marburg, Georg-Voigt-Str. 3, 35039 Marburg, Germany

**Keywords:** Orthodontic treatment, Tooth enamel, Fixed orthodontic appliances, Quantitative light-induced fluorescence, Dental white spots, Kieferorthopädische Behandlung, Zahnschmelz, Festsitzende kieferorthopädische Apparaturen, Quantitative lichtinduzierte Fluoreszenz, White Spots

## Abstract

**Purpose:**

Enamel demineralization can occur as a side effect during orthodontic treatment with fixed appliances and should be detected as early as possible. A new approach to assess demineralization is a system consisting of a photosensitive protein that binds to free calcium ions at the enamel surface. A camera is then used to visualize the bioluminescence spots. This in vitro study aimed to evaluate the ability of the bioluminescence technology to assess artificially demineralized enamel adjacent to various orthodontic brackets.

**Methods:**

In all, 108 human enamel samples were allocated randomly to groups with different orthodontic bracket material: stainless steel, titanium, ceramic. Initial lesions were created adjacent to the brackets. The samples were assessed by bioluminescence before and after demineralization. Images were assessed for presence of bioluminescence spots (yes/no). To quantify the bioluminescence measurements, the images’ pixel values (P) were calculated within a defined area (F) adjacent to each bracket before and after demineralization. Quantitative light-induced fluorescence measurements (ΔF, ΔQ) were performed as the reference standard for demineralization.

**Results:**

After demineralization, bioluminescence spots were visible (yes/no decision) in 87% of the samples. The pixel analysis of the bioluminescence spots showed significantly higher pixel values after demineralization compared to baseline (*p* < 0.0001). The bracket material had no influence on the bioluminescence measurements. All samples showed fluorescence loss with a median ΔF of −9.52% (±3.15) and a median ΔQ of −1.01% × mm^2^ (±3.34), respectively.

**Conclusion:**

The bioluminescence technology is a promising tool to demonstrate demineralization adjacent to different orthodontic brackets in vitro.

## Abstract (de)

**Zielsetzung:**

Schmelzdemineralisationen können während einer kieferorthopädischer Behandlung mit festsitzenden Apparaturen auftreten und sollten so früh wie möglich erkannt werden. Ein neuer Ansatz ist ein System basierend auf einem lichtsensitiven Protein, das freie Kalziumionen an der Schmelzoberfläche bindet und Biolumineszenzfelder visualisiert. Das Ziel dieser In-vitro-Studie war es, die Eignung der Biolumineszenztechnologie zur Detektion von artifiziell erzeugten Demineralisationen im Schmelz in der Zirkumferenz von verschiedenen kieferorthopädischen Brackets zu untersuchen.

**Methode:**

Insgesamt 108 humane Zähne wurden randomisiert in Gruppen mit unterschiedlichem kieferorthopädischem Bracketmaterial eingeteilt: Edelstahl, Titan, Keramik. Am Rand der Brackets wurden Initialläsionen erzeugt. Die Proben wurden vor und nach der Demineralisation mittels Biolumineszenz untersucht. Die Bilder wurden auf das Vorhandensein von Biolumineszenzfeldern (ja/nein) bewertet. Zur Quantifizierung der Biolumineszenzmessungen wurden die Pixelwerte (P) der Bilder innerhalb eines definierten Bereichs (F) am Rand der Brackets vor und nach der Demineralisation berechnet. Quantitative lichtinduzierte Fluoreszenzmessungen (ΔF, ΔQ) wurden als Referenzstandard für die Demineralisation herangezogen.

**Ergebnisse:**

Nach der Demineralisation waren bei 87 % der Proben Biolumineszenzfelder sichtbar (Ja/nein-Entscheidung). Die Analyse der Biolumineszenzfelder zeigte signifikant höhere Pixelwerte nach der Demineralisation im Vergleich zu den Werten vor der Demineralisation (*p* < 0,0001). Das Bracketmaterial hatte keinen Einfluss auf die Biolumineszenzmessungen. Alle Proben zeigten einen Fluoreszenzverlust mit einem ΔF-Medianwert von −9,52 % (± 3,15) sowie einem ΔQ-Medianwert von −1,01 % × mm^2^ (±3,34).

**Schlussfolgerung:**

Die Biolumineszenztechnologie hat sich in vitro als ein vielversprechendes Verfahren zur Visualisierung von Demineralisationen in der Bracketzirkumferenz erwiesen.

## Introduction

Orthodontic treatment with fixed multibracket appliances is a common method to treat malpositioned teeth. A standard procedure is to attach brackets on the labial or lingual surfaces of the teeth. Treatment with multibracket appliances makes oral hygiene more difficult and leads to undesired adverse effects to the teeth and the surrounding structures [28]. Increased plaque accumulation around the brackets can enhance the development of demineralization adjacent to the brackets [27]. Without proper preventive intervention, such lesions can progress to dentine caries [11].

Early detection of demineralization adjacent to brackets is challenging. Although visual assessment is an important method for detecting initial lesions, it is often subjective; thus, subsidiary methods may provide additional benefit in objectifying and documenting demineralization, including digital documentation. Furthermore, remineralizing measures can only be used effectively if demineralization is detected early and the evident success of remineralizing procedures, also in terms of follow-up, can significantly improve patient cooperation.

The recently introduced Calcivis® Imaging System (Calcivis Ltd, Edinburgh, UK) is a method based on the bioluminescence technique for detecting demineralization in enamel. It is known that during demineralization of the enamel, free calcium ions are released [18, 33]. The bioluminescence technique consists of applying a specific calcium-sensitive photoprotein to the tooth surface. The photosensitive protein binds to these calcium ions and a light signal is emitted upon binding to solvated calcium ions. If the tooth is undergoing net demineralization a light signal can be observed [19]. Such luminescence areas are typically present as blue spots demonstrating the presence of demineralized surfaces.

To date, only a few studies have been performed to detect active caries lesions and distinguish them from sound or inactive lesions [8, 16, 17]. However, these studies were performed on the occlusal surfaces without any interaction with other materials. No published data are available in which the bioluminescence system is used to evaluate demineralization adjacent to brackets. Therefore, the aim of this study was to investigate whether artificially induced demineralization around orthodontic brackets can be visualized by the bioluminescence technology. Different bracket types were used to assess whether the bracket material has an influence on the bioluminescence measurements. The quantitative light-induced fluorescence technology (QLF) served as the reference standard to evaluate the demineralization procedure.

## Materials and methods

### Selection of extracted teeth

The use of extracted human teeth was approved by the ethics committee of the medical faculty of the Philipps University of Marburg (reference no. 132/19). A sample size calculation was performed using the software G*Power, V 3.1.9.2 [12]. Based on the preliminary ascertained data, a high correlation of 0.7 was assumed between bioluminescence measurements and the reference standard histology. Thus, 34 samples were calculated to be included in each bracket group (power 0.95, α = 0.05). A drop-out number of two samples per group was added.

The teeth were stored after extraction in a 0.001% sodium azide solution for disinfection. In case of presence of adherent soft tissues they were carefully removed. According to the protocol of the manufacturer of the bioluminescence device the teeth were cleaned with a cleaning paste with an RDA (relative dentin abrasivity) value of 120 (Clinpro Prophy Paste, 3M ESPE, Seefeld, Germany) applied with a rotating white cup brush with nylon filaments (Pluradent GmbH, Offenbach, Germany). The remaining paste was removed with a multifunctional syringe using water and air, and the teeth were stored in deionized water afterwards. The buccal surfaces of the teeth were examined under a stereomicroscope (Leica MS 5, Leitz, Wetzlar, Germany) at ×16 magnification. Care was taken to ensure that every surface was without signs of demineralization or enamel defects. Then, the samples were fixed on plexiglas slides (Dia-plus, Oststeinbek, Germany).

On each sample (buccal site of the teeth) orthodontic brackets were bonded following the instructions for use: the enamel surface was etched (36% phosphoric acid gel: Conditioner 36, Dentsply DeTrey, Konstanz, Germany) and rinsed with water after 30 s. The teeth were dried and orthodontic brackets were bonded in the center of each sample (Transbond XT primer and adhesive, 3M Unitek, Landsberg, Germany) and were light cured (FlashMax P4 Ortho Pro, orthodontic light pen, CMS Dental, Copenhagen, Denmark). Three different bracket types (Dentaurum, Ispringen, Germany) were used to evaluate whether the material (stainless steel, titanium or ceramic) has an influence on the bioluminescence measurements: group A = equilibrium® mini (material: stainless steel); group B = equilibrium ti® (material: titanium); and group C = discovery® pearl (material: ceramic composed of aluminum oxide crystals).

### Demineralization of the samples

To create an internal control group, one side of each sample was covered by an acid resistance clear nail polish (Manhattan, Mainz, Germany). Hence, this site was protected from demineralization. To produce initial enamel lesions, the samples were covered with a layer of an 8% methylcellulose (approximately 2 cm) on top of which 0.1 M lactate buffer was placed in excess at a pH of 4.6 [29] at 37 °C (incubator type B, Heraeus GmbH, Hanau, Germany). The samples were removed after 14 days. After rinsing and air drying all surfaces were examined under a microscope to check for a whitish, dull surface typically for initial enamel lesions.

### Bioluminescence measurements

A bioluminescence system for in vitro application was used for quantification of free available calcium on the tooth surfaces according to manufacturer’s operating manual. The methodology was already published for use in occlusal surfaces [17]. In brief, a camera in the box was connected to a laptop and an applicator needle (integrated in the box) was connected to a pipette outside of the box. A solution was prepared mixing a freeze-dried protein powder with distilled water in a predetermined concentration and was transferred to the pipette. The sample was placed under the camera and the solution (100 µl for each surface) was applied to the sample surface through the pipette. A grey luminescent image was displayed on the screen and this image was processed with the image analysis software ImageJ (Fiji) to display a colored luminescent image.

The bioluminescence measurements were performed at baseline and after demineralization. The images were examined for luminescence areas on the surface which correspond to freely available calcium. First, yes/no decisions were made for the presence of luminescence spots on the images. Then, the luminescence area was marked and the number of the colored pixels in the area was assessed. Mean pixel values and the area of the luminescence spots in the areas of interest were calculated and compared prior to and after demineralization.

### Quantitative Light-induced Fluorescence (QLF^TM^)

As a nondestructive method, QLF measurements were performed to quantify the demineralization (QLF Inspektor Pro, Inspektor Research Systems, Amsterdam, The Netherlands with the software Inspektor Pro 2.0.0.48). The measurements were performed at baseline and after demineralization. Average percentage of fluorescence loss with respect to the fluorescence of sound tissue (ΔF in %, related to lesion depth) and fluorescence loss times the area (ΔQ in % × mm^2^, related to lesion volume) were analyzed [34].

### Statistical evaluation

The statistical evaluation was performed using the software MedCalc® (v19.2.1). Data were tested for normal distribution using the Shapiro–Wilk’s test (*p* < 0.05) and nonparametric tests were used for further analysis (Wilcoxon test, Kruskal–Wallis test). McNemar test was used to evaluate the differences in the Calcivis readings (luminescence spots yes/no). The significance level was set at α = 0.05.

## Results

A total of 108 samples were included in the study. At baseline, no luminescence spots were detectable on the samples, indicating there was no demineralization of the surfaces. After demineralization, 87% of the samples showed luminescence spots (McNemar test, *p* < 0.001) representing demineralization areas adjacent to the brackets (Table 1).

**Table 1:** Cross tabulation of number of the samples with and without bioluminescence spots prior and after demineralization Kreuztabellierung der Proben mit und ohne Biolumineszenz-Spots vor und nach Demineralisierung

**Bracket group A: stainless steel**	*Bioluminescence after demineralization, n (%)*				
*Bioluminescence at baseline*	*No*	*Yes*		*Total*	
*No*	6 (16.7%)	30 (83.3%)		36 (100%)	
*Yes*	0 (0%)	0 (0%)		0 (0%)	
*Total*	6 (16.7%)	30 (83.3%)		36 (100%)	
**Bracket group B: titanium**	*Bioluminescence after demineralization, n (%)*				
*Bioluminescence at baseline*	*No*	*Yes*		*Total*	
*No*	2 (5.6%)	34 (94.4%)		36 (100%)	
*Yes*	0 (0%)	0 (0%)		0 (0%)	
*Total*	2 (5.6%)	34 (94.4%)		36 (100%)	
**Bracket group C: ceramic**	*Bioluminescence after demineralization, n (%)*				
*Bioluminescence at baseline*	*No*	*Yes*		*Total*	
*No*	6 (16.7%)	30 (83.3%)		36 (100%)	
*Yes*	0 (0%)	0 (0%)		0 (0%)	
*Total*	6 (16.7%)	30 (83.3%)		36 (100%)	

Software analysis of the luminescence spots showed significantly higher pixel values and areas after demineralization (Wilcoxon test, *p* < 0.0001). The luminescence spots between the different bracket groups did not differ significantly after demineralization (Kruskal–Wallis test, pixel values: *p* = 0.65; pixel × area: *p* = 0.18). The pixel values of the bioluminescence areas in each bracket group are summarized in Table 2.

**Table 2:** Results of the bioluminescence measurements (pixel number in the bioluminescence areas) in each bracket group Ergebnisse der Biolumineszenzmessungen (Pixelzahl in den Biolumineszenzfeldern) in jeder Bracketgruppe

	Min	Max	Mean	Median	SD	*p*-value
*Bracket group A: stainless steel*						
Pixels at baseline	0.60	4.12	1.22	0.60	0.85	<0.0001
Pixels after demineralization	0.69	25.55	7.01	6.11	5.10	
Pixels × area at baseline	448.80	12,877.98	3004.39	2275.24	2961.63	<0.0001
Pixels × area after demineralization	562.38	62,585.25	16,925.43	15,260.13	15,134.55	
*Bracket group B: titanium*						
Pixels at baseline	0.64	3.67	1.10	0.82	0.71	<0.0001
Pixels after demineralization	0.97	15.95	7.21	6.01	3.42	
Pixels × area at baseline	564.82	22,992.09	4349.77	2581.77	5198.45	<0.0001
Pixels × area after demineralization	1615.41	93,788.16	25,384.89	18,925.69	22,029.19	
*Bracket group C: ceramic*						
Pixels at baseline	0.65	7.22	1.55	0.95	1.33	<0.0001
Pixels after demineralization	2.17	22.97	7.52	6.75	4.33	
Pixels × area at baseline	524.66	33,497.64	4634.84	2632.02	6326.58	<0.0001
Pixels × area after demineralization	1143.12	97,513.88	22,384.95	16,041.29	21,363.11	

*SD* standard deviation, *min* minimum, *max* maximum

After demineralization, all samples showed a distinct fluorescence loss with a median ΔF of −9.52% (±3.15) and a median ΔQ of −1.01% × mm^2^ (±3.34), respectively. There were no significant differences between the bracket groups after demineralization (Kruskal–Wallis test, ΔF: *p* = 0.44; ΔQ: *p* = 0.36). The values for the percentage of fluorescence loss (ΔF and ΔQ) in each bracket group are summarized in Table 3.

**Table 3:** Results of the quantitative light-induced fluorescence (QLF) measurements in each bracket group after demineralization Ergebnisse der QLF(quantitative lichtinduzierte Fluoreszenz)-Messungen in jeder Bracketgruppe nach Demineralisation

	Min	Max	Mean	Median	SD
*Bracket group A: stainless steel*					
ΔF	−21.10	−6.90	−10.37	−9.44	3.61
ΔQ	−17.70	−0.10	−3.16	−1.34	4.22
*Bracket group B: titanium*					
ΔF	−18.60	−6.85	−10.61	−9.70	3.10
ΔQ	−8.66	−0.03	−1.46	−0.61	1.91
*Bracket group C: ceramic*					
Pixels	−17.70	−6.97	−9.49	−8.74	2.50
Pixels × area	−12.20	−0.010	−2.17	−1.05	3.02

*ΔF* fluorescence loss (%), *ΔQ* fluorescence loss × area (% × mm^2^), *SD* standard deviation, *Min* minimum, *Max* maximum

**Fig. 1:**
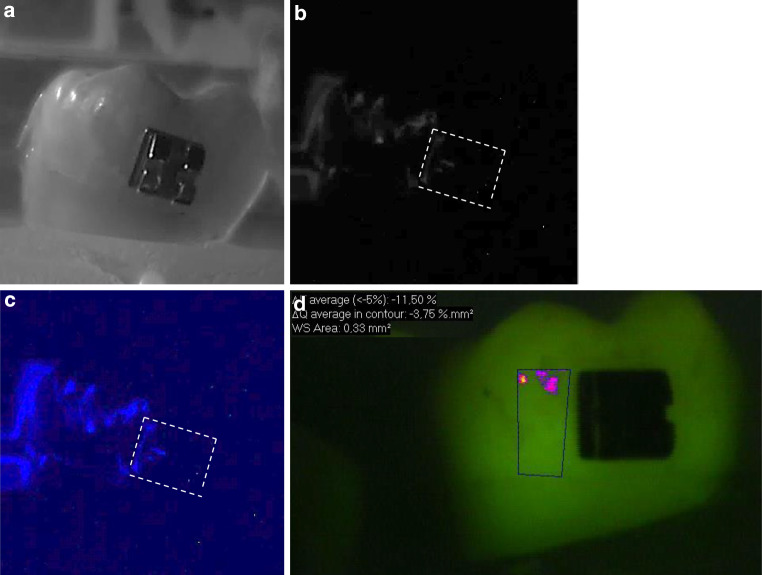
Representative sample of bracket group A (stainless steel) after demineralization. The *left side* of the samples was exposed to the demineralized solution. The *white dashed line* shows the position of the bracket. **a** Standard visible image, **b** corresponding grey bioluminescence image, **c** corresponding colored bioluminescence image, **d** corresponding quantitative light-induced fluorescence (QLF) image Repräsentative Probe der Bracketgruppe A (Edelstahl) nach der Demineralisierung. Die *linke Seite* der Proben wurde demineralisiert. Die *weiße gestrichelte Linie* zeigt die Position des Brackets. **a** Sichtbares Standardbild, **b** korrespondierendes graues Biolumineszenzbild, **c** korrespondierendes gefärbtes Biolumineszenzbild, **d** korrespondierende QLF(quantitative lichtinduzierte Fluoreszenz)-Aufnahmen

**Fig. 2:**
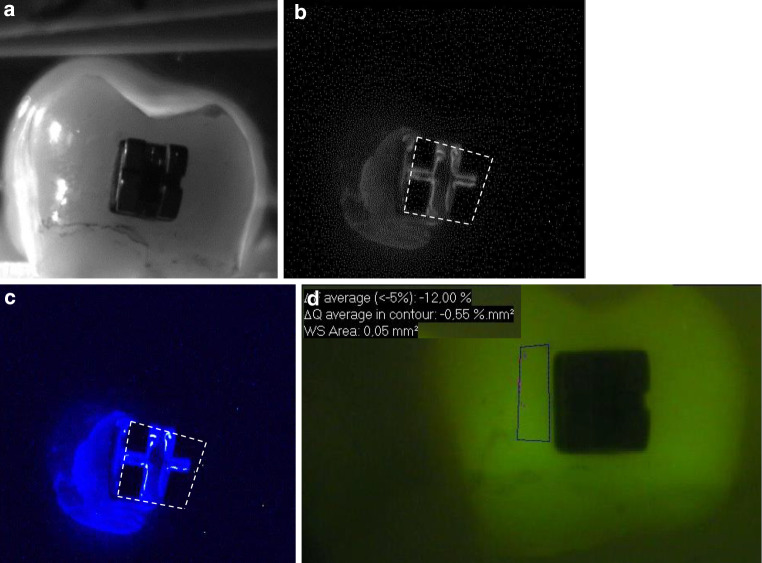
Representative sample of bracket group B (titanium) after demineralization. The *left side* of the samples was exposed to the demineralized solution. The *white dashed line* shows the position of the bracket. **a** Standard visible image, **b** corresponding grey bioluminescence image, **c** corresponding colored bioluminescence image, **d** corresponding quantitative light-induced fluorescence (QLF) image Repräsentative Probe der Bracketgruppe B (Titan) nach der Demineralisierung. Die *linke Seite* der Proben wurde demineralisiert. Die *weiße gestrichelte Linie* zeigt die Position des Brackets. **a** Sichtbares Standardbild, **b** korrespondierendes graues Biolumineszenzbild, **c** korrespondierendes gefärbtes Biolumineszenzbild, **d** korrespondierende QLF(quantitative lichtinduzierte Fluoreszenz)-Aufnahmen

**Fig. 3:**
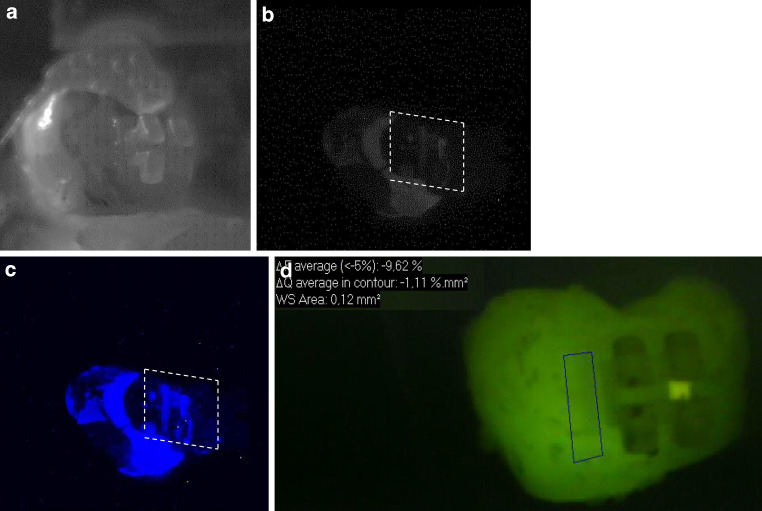
Representative sample of bracket group C (ceramic) after demineralization. The *left side* of the samples was exposed to the demineralized solution. The *white dashed line* shows the position of the bracket. **a** Standard visible image, **b** corresponding grey bioluminescence image, **c** corresponding colored bioluminescence image, **d** corresponding quantitative light-induced fluorescence (QLF) image Repräsentative Probe der Bracketgruppe C (Keramik) nach der Demineralisierung. Die *linke Seite* der Proben wurde demineralisiert. Die *weiße gestrichelte Linie* zeigt die Position des Brackets. **a** Sichtbares Standardbild, **b** korrespondierendes graues Biolumineszenzbild, **c** korrespondierendes gefärbtes Biolumineszenzbild, **d** korrespondierende QLF(quantitative lichtinduzierte Fluoreszenz)-Aufnahmen

In Figs. 1, 2 and 3, representative images of each bracket group are presented.

## Discussion

Enamel demineralization is one of the most undesired side effects of fixed orthodontic treatment. High treatment demand and occurrence of biofilm-related complications requiring professional care were named to make orthodontic treatment a potential public health threat [25]. In order to manage this problem, the first approach would be to detect such initial lesions and assess them for presence of activity signs. In the next steps, the caries risk can be modified and emphasis on management strategies which focus on remineralization of the lesions can take place.

The detection of demineralization at sites that actively lose mineral content at an early stage is challenging. Visual–tactile examination has been established for the detection of active caries lesions [9, 22], although there is a margin for error due to the relative subjectivity of the method. This may lead to many patients not receiving preventive treatment to reverse the demineralization process or, on the other hand, some who receive unnecessary treatment on inactive sites. The use of magnification aids can be discussed for more accurate detection of early enamel lesions; however, this is not a standardized clinical procedure and can lead to low specificity values [20]. It was reported that magnification would not improve the accuracy of visual scoring systems in the detection of occlusal caries lesions [35]. Furthermore, there are reports of using optical devices for detection and quantifying of noncavitated early enamel lesions [14] as a tool for the documentation and monitoring of lesions. Such methods can support the diagnostic process in quantifying and/or visualizing caries lesions. A new approach for detecting enamel demineralization is the assessment of free calcium ions indicating enamel demineralization. In a preliminary study on occlusal caries lesions, the bioluminescence system demonstrated high reproducibility and good diagnostic accuracy values for the assessment of active caries lesions in vitro [17] based on histology. Another study showed lower sensitivity and specificity values for the bioluminescence system compared to the fluorescence-based system when active initial lesions were detected on occlusal surfaces [8]. However, the results were based on visual assessment of the teeth as a reference, so that the results cannot be compared to the study by Jablonski-Momeni et al. [17] due to the lack of histological findings. Longbottom et al. [19] used the bioluminescence technology for assessment of artificially produced demineralization in dental enamel at different pH values such as acidic solutions. The lower the pH of the solution, the greater was the light signal recorded, thus, showing the potential of the technology to demonstrate enamel erosion.

The present study was the first to evaluate the ability of the system to assess demineralization on smooth surfaces adjacent to orthodontic brackets. The results indicate the suitability of the bioluminescence technique to visualize artificially demineralized areas. A shortcoming of the bioluminescence system is the lack of any scale for quantification of demineralized areas. Currently, only a yes/no decision can be made with regard to the presence of luminescence spots, and the assessment of the colored bioluminescence spots was much easier on the computer screen than on printed images. Thus, in order to quantify the findings, an attempt was made to represent the measurements in numbers by calculating the pixel values in the luminescence spots using image software. The results show that luminescence spots were detectable after demineralization (yes/no decision) and that significantly higher pixel values were measured after demineralization of the enamel compared to the values from baseline measurements (Table 2). However, further developments are required to quantify the extent and the area of demineralization directly with the bioluminescence system.

Looking at Figs. 1, 2 and 3, it is obvious that not all areas of the demineralized sample region clearly showed colored spots. This could be due to the fact that the samples were buccal surfaces of human teeth and the surfaces were not completely flat. Moreover, the brackets may have prevented the adjacent area from being coated by the photoprotein. Therefore, the material could not sprinkle the complete sample surface. In contrast, studies on occlusal lesions showed more clearly bioluminescence spots in pits and fissures [17]. Interference of the bonding material on the bioluminescence measurements can also be assumed. Staudt et al. [26] showed that after bonding with Transbond XT primer and adhesive, fluorescence values decreased compared to the prebonded and etched enamel. Other authors showed that the use of a resin composite bonding system with the ability of fluoride release for bracket bonding may reduce demineralization of enamel around brackets during orthodontic treatment [24]. In our study we used a bonding material without additional fluoride release, so only little influence of the material on the measurements would be expected.

In the present study, the quantity of luminescence spots between the different brackets groups did not differ significantly, indicating that the presence of brackets would not affect the bioluminescence measurements. Some studies demonstrated no differences in the adherence of cariogenic streptococci to stainless steel, ceramic, or plastic brackets [4, 21]. On the other hand, some authors reported higher bacterial attachment rates in ceramic or plastic brackets compared to metal brackets [1, 23, 31]. In our study, a demineralization model without colonization of cariogenic microorganisms was used and therefore this factor could not be taken into consideration. In a study where enamel was demineralized artificially, it was shown that teeth bonded with ceramic brackets showed significantly higher enamel demineralization compared to teeth bonded with metal brackets [3]. In a systematic review it was concluded that there is currently no evidence for a possible influence of the design of the brackets (conventional or self-ligating) over colony formation and adhesion of *Streptococcus mutans* and that other factors such as the quality of the bracket type, the level of individual oral hygiene, bonding and age may have greater influence [10].

The use of QLF is a common method to assess demineralization adjacent to orthodontic brackets [5–7, 13, 30, 32]. In QLF measurements, fluorescence is correlated with the mineral loss of a tooth surface [15]. Thus, the characteristics of this method make it suitable for monitoring of mineral changes in initial enamel lesions and also for the evaluation of preventive measures [2].

## Conclusions

In the present study, the ability of the bioluminescence technique to detect artificially produced demineralization adjacent to orthodontic brackets was demonstrated. At the time when our study was performed, only a device for in vitro use was available. Recently, the bioluminescence technology was introduced for in vivo detection of active demineralization and the findings of our study can be transferred to the clinical situation in the near future. Based on the results and within the limitations of an in vitro study, the conclusion can be drawn that demineralization adjacent to orthodontic bracket of any material can be demonstrated digitally by means of bioluminescence. This allows a more objective assessment of active demineralization compared to visual detection and monitoring of tooth hard tissue, for example, in adolescents undergoing orthodontic treatment.

In further studies, the performance of the bioluminescence method to assess demineralization should be evaluated clinically. In addition, studies are needed to evaluate the ability of the bioluminescence method to also assess remineralization of enamel using different remineralizing agents.
